# Forecast the Exacerbation in Patients of Chronic Obstructive Pulmonary Disease with Clinical Indicators Using Machine Learning Techniques

**DOI:** 10.3390/diagnostics11050829

**Published:** 2021-05-04

**Authors:** Ali Hussain, Hee-Eun Choi, Hyo-Jung Kim, Satyabrata Aich, Muhammad Saqlain, Hee-Cheol Kim

**Affiliations:** 1 Institute of Digital Anti-Aging Healthcare, Inje University, Gimhae 50834, Korea; alihussain.dream@gmail.com (A.H.); satyabrataaich@gmail.com (S.A.); 2 Department of Physical Medicine and Rehabilitation, Haeundae Paik Hospital, Inje University College of Medicine, Busan 48108, Korea; solideogloria@paik.ac.kr; 3 Department of Internal Medicine, Haeundae Paik Hospital, Inje University College of Medicine, Busan 48108, Korea; khj850819@naver.com; 4 Department of Computer Science & Engineering, Ulsan National Institute of Science and Technology, Ulsan 44919, Korea; m.saqlain1240@yahoo.com; 5 College of AI Convergence/Institute of Digital Anti-Aging Healthcare/u-HARC, Inje University, Gimhae 50834, Korea

**Keywords:** chronic obstructive pulmonary disease (COPD), machine learning, features set, disease severity, prediction models

## Abstract

Preventing exacerbation and seeking to determine the severity of the disease during the hospitalization of chronic obstructive pulmonary disease (COPD) patients is a crucial global initiative for chronic obstructive lung disease (GOLD); this option is available only for stable-phase patients. Recently, the assessment and prediction techniques that are used have been determined to be inadequate for acute exacerbation of chronic obstructive pulmonary disease patients. To magnify the monitoring and treatment of acute exacerbation COPD patients, we need to rely on the AI system, because traditional methods take a long time for the prognosis of the disease. Machine-learning techniques have shown the capacity to be effectively used in crucial healthcare applications. In this paper, we propose a voting ensemble classifier with 24 features to identify the severity of chronic obstructive pulmonary disease patients. In our study, we applied five machine-learning classifiers, namely random forests (RF), support vector machine (SVM), gradient boosting machine (GBM), XGboost (XGB), and K-nearest neighbor (KNN). These classifiers were trained with a set of 24 features. After that, we combined their results with a soft voting ensemble (SVE) method. Consequently, we found performance measures with an accuracy of 91.0849%, a precision of 90.7725%, a recall of 91.3607%, an F-measure of 91.0656%, and an AUC score of 96.8656%, respectively. Our result shows that the SVE classifier with the proposed twenty-four features outperformed regular machine-learning-based methods for chronic obstructive pulmonary disease (COPD) patients. The SVE classifier helps respiratory physicians to estimate the severity of COPD patients in the early stage, consequently guiding the cure strategy and helps the prognosis of COPD patients.

## 1. Introduction

Recently, machine-learning techniques have revolutionized the entire technological domain. The machine-learning technique (ML) is considered as a subset of artificial intelligence (AI). Ordinarily, these types of intelligence are mostly acknowledged as having initiated with the invention of robotics [1]. With the fast growth of programming and electronic speeds, in the near future, computers may be able to display intelligent behaviors the way humans do [2]. AI can be interpreted as the human brainpower which performs by machine. In the field of computer science, it is explained as the machine’s ability to learn by itself for imitating intelligence behavior [3]. Due to the revolution in the computer, every field takes benefit from this innovation, and the medical sector is also one of those areas which are making advancement with the help of latest technologies, such as AI. Machine learning (ML) took a significant place in medical diagnosis because of its better classification with high accuracy rates.

Chronic obstructive pulmonary disease (COPD) is an obstructive kind of lung disease. COPD disease has a characteristically bad impact on our respiratory system. This disease characteristically tangles an airflow limitation that is not completely reversible. COPD is an ordinary consequence for subjects exhibiting suffering from chronic bronchitis or emphysema. In the situation of emphysema, the alveoli at the finale of the bronchioles (tiniest air passages of the lungs) are ruined, normally from smoking.

Daily cough and phlegm production are the major signs of chronic bronchitis, which is of a minimum duration of three months a year. The pithiness of breath, wheezing, chest tightness, and chronic cough are the main vital signs of COPD disease. COPD disease is not fully reversible, although proper cure guidance and therapy can slow its infection, minimize its complexity, and help to improve the quality of life of patients [4]. The avoidance of acute exacerbation is the major problem that leads to the fast worsening of a patient’s health condition [5]. Now, here we discuss the exacerbation. Exacerbation does not even have a clear definition yet, because the good relationship between danger factors and the progress of exacerbations is not completely understood [6]. Chronic obstructive pulmonary disease (COPD)-affected people are increasing compared to the past. It may be increasing at a higher rate because our population growth rate increasing over time. It is a global threat, so we need to take action to reduce it; otherwise, it will become the third major cause of death in the world by 2030 [7]. Howbeit, the morbidity and mortality of COPD patients can be reduced, but it is only possible when finding the disease in the early stage [8,9]. In 2012, 3 million people died due to chronic obstructive pulmonary disease (COPD), and this amount is equivalent to 6% of the entire death of that year [10]. Even though pharmacotherapies have improved for COPD, innumerable patients still experience an exacerbation of COPD, during which respiratory signs worsen acutely, and which ascertain disease-associated morbidity rate, mortality rate, resource burden, and healthcare expenses [11]. As reported by the world economic forum (WEF) in 2030, the expenses associated with chronic disease may reach 47 trillion globally. In the past few years, consumption of tobacco has increased; only awareness and proper strategy can reduce its consumption. COPD is ordinarily due to cigarette smoking, even though long-term exposure to other lung nuisances, such as passive tobacco smoke, can also enhance COPD [12,13]; however, in COPD, the major cause is smoking [14]. So far, much research has relied on cohort data for the prognosis of COPD exacerbation. Furthermore, most of them have focused on associated internal factors intrinsic to the COPD victim themselves, instead of external factors [15,16,17]. Moreover, we know that external factors are also contribute to enhancing the exacerbation of COPD patients, such as viral infection and air pollution [18,19,20]. In recent years, supervised machine learning and unsupervised machine learning are working successfully in the field of autonomous diagnosis of many diseases. In this paper, we used a supervised machine-learning technique to seek the severity in COPD patients. Machine learning (ML) models are used to determine the complex problem by extracting medical information; they change the novel ideas into real-time for practitioners and professionals [2]. In real-time clinical practice, models can also perform in decision-making for individual patient cures. These models have a capacity for autonomous diagnosis of many diseases beneath clinical regulations [21,22,23,24]. Moreover, using ML models improves the quality, and minimizing the fluctuations in patients’ rates of medical data can be possible, saving medical expenditure. In the past, the classical machine-learning techniques such as RF, SVM, GBM, XGB, and KNN have been used successfully in the healthcare sector. Aich et al. proposed a framework that used SVM, KNN, DT, and NB for classification of two groups of Parkinson’s disease (PD) patients, using the data from the wearable devices [25]. Chang et al. proposed a solution that used RF, DT, XGB, and SVM for the prediction of hypertension outcomes, using medical data [26]. Abedi et al. proposed a framework that used LR, XGB, GBM, SVM, RF, and DT for the prediction of long-term stroke recurrence [27]. Aich et al. proposed a solution that used SVM, RF, and NB for classifying two groups, namely PD patients with shuffling of gaits and other old adults [28]. Aich et al. also proposed a framework that used KNN, SVM, NB, and DT for distinguishing PD patients from a healthy group of patients [29]. Based on the results of the past studies, we have included these classifiers for our studies. To improve the performance of our proposed framework, we have decided to use an ensemble method to get best out of all of them, instead of using a single classifier for our study. So, we have used the ensemble classifier technique, which finds out the best from the individual classifier, to make a robust model for our proposed system. Moreover, the ensemble classification technique has become a famous topic in the domain of ML and is used to control the limitation of independent classifiers [30]. Ensemble methods aim to integrate the different ML models’ predictions which have different learning parameters and collect the final prediction result with high accuracy. Ensemble classifiers have shown more effective results about the stability and robustness as compared to individual classifier’s performance [31]. The ensemble method decreases the issue of over-fitting and under-fitting during the training and validation [32]. The ensemble system stands on three basic pillars, namely diverseness, the training of every classifier that is part of ensemble system, and integrating the results of all member of classifiers, using weighted majority voting or simple voting to get and combined the result [33]. The performance of the ensemble system depends on the performance of the individual classifiers. If we include more classifiers, the ensemble system will perform better. However, the selection of felicitous classifiers for preparing the ensemble system remains a very arduous topic. Moreover, we know that the performance of all classifiers cannot similar, because every classifier has its parameters and regulations to perform on the dataset. All classifiers cannot well recognize all classes; for instance, one classifier can do well identifying the mild class, whereas another classifier can do well at identifying the severe class. However, the ensemble system of these classifiers will perform precisely to identify both classes. Machine-learning methods have been adequately utilized in the computerized elucidation of pneumonic capability tests for the different analyses of chronic diseases. It is anticipated that the models with supreme accuracies could get huge significance in medical diagnosis. It was found that not enough studies using extensive number of features using machine-learning techniques have been conducted in the past. Although there were few studies discussed about the detection of COPD, those studies were not extensive for use in the real-time application.

Therefore, in this research work, we propose a voting ensemble classifier to identify the mild and severe classes in chronic obstructive pulmonary disease (COPD) patients for medical prognosis with high accuracy. Characteristically, chronic obstructive pulmonary disease (COPD) is a naturally slow-progressing disease, so it is crucial make an initial stage prediction and provide effective medication. It is necessary to propose an ensemble classifier that can help to diagnose COPD disease in a precise manner and predict coming time patient outcomes. There are many ways to approach AI, one of them is an ensemble method. The current study focuses clearly on predictive models utilized in the diagnosis of COPD that illuminate the importance of this work. We used a dataset including twenty-nine hundred patients who were recruited at Inje university Haeundae Paik Hospital, Korea. There are two different kinds of patients in this dataset, one is a mild- and the other is a severe-condition patient.

In the beginning, the dataset containing two groups of patients, namely mild- and severe-condition patients, had 54 features; considering the demand for the reliable and fast system we used feature-selection techniques in conjunction with the opinion of the experts in these field. By using the RFE feature-selection technique, we reduced to the features to 30. Then, after consulting with a physician/doctor, we reduced to features to 24. We found that the two group contains imbalanced data. Since the imbalanced dataset is a common issue mostly in medical data, the synthetic minority oversampling technique (SMOTE) algorithm was used to up-sample the data [34]. In the next step, we implemented five different classifiers, namely RF, SVM, GBM, XGB, and KNN, to train the models. Then we used a soft voting ensemble (SVE) approach to combine the results. Lastly, we calculated the performance measures of the models. For instance, accuracy, precision, recall, f-measure, and area under ROC curve (AUC) were used to acquire the final classification. The result of the proposed ensemble classifier shows that it will be helpful to attain the severity assessment of disease in COPD patients to help the physicians after the patients’ hospital admission.

The organization of the below parts of this paper is as follows: Section 2 pertains to the related work in line with this research paper. Section 3 discusses the data collection and also the methods used in this research. Section 4 contains the results of the proposed method. Section 5 and Section 6 present the discussion and conclusion of this research paper, respectively.

## 2. Related Work

Many researchers in the past few years have investigated the analysis of COPD patient’s conditions and have tried to explore the exacerbation, and they used different methodologies to point out the exacerbation. Some previous related research works are mentioned below.

Peng et al. proposed a method using the C5.0 decision tree classifier with 28 features, in which medical history, comorbidities, and other various inflammatory and vital sign indicators were selected. The proposed method was developed to seek the severity (mild and severe) of disease in COPD patients. They applied different classifiers but found C5.0 classifier performs well and found 80.3% accuracy. For this study, 410 patient data were used [35]. COPD patients need help in everyday life to avoid exacerbation. They need a daily life monitoring system to protect the frequent risk of acute exacerbation to control their disease stage. An automated monitoring system could guide them to get appropriate treatment and avoids gratuitous hospital (re-)admission. Nunavath et al. proposed two deep-learning approaches, firstly feed-forward neural networks (FFNN) were used for classification of patient’s category, and the second long short -term memory (LSTM) was used for early prediction of COPD exacerbation and subsequent triage. The data collected from the family environment is not considered to be good, they can interfere with various factors, leading to the worsening of data quality. They found that the FFNN model classified COPD patients with 92.86% accuracy, and the LSTM model predicted the patient’s health condition with 84.12% accuracy [36]. Siddhi and Chintan proposed SVM (Support Vector Machine) and KNN (K-nearest neighbor) to check the COPD patient’s disease level. The kernel choice was not a wholly solved issue, but they observed that the linear kernel was good in that case. They have found classification with 96.97% accuracy using SVM and with KNN 92.30% accuracy. They observed this method to help assist the doctor to determine the level of COPD patients more quickly [37]. Fernandez-Granero et al. proposed an approach that was able to automatically detect early severity in COPD patients using respiratory sound. They recorded respiratory sounds daily using a sensor, after that, they designed a decision tree forest classifier (DTF) and found an accuracy of 75.8% [38]. Amaral et al. proposed artificial neural networks (ANNs) for the diagnosis of COPD patients, forced oscillation measurements (FOT) method is used for the collection of data. They tried to find the utmost crucial parameters and also reduced the dataset. For this purpose, they have used two feature selection methods. They have found an accuracy of more than 90%, a sensitivity of more than 90%, and an AUC value of more than 90% [39]. Archana and V.K proposed a Support vector machine (SVM) algorithm that could separate COPD patients from normal subjects using electromyography (inhalation and exhalation progression), separated COPD patients from normal subjects with an accuracy of 85% [40]. FANG et al. proposed a method to integrate the model based on a knowledge graph for diagnosing COPD. The first step created a knowledge graph and found the relationship between feature sets, and then further tried to find the knowledge of implicit disease from the data. Secondly, they proposed the algorithm CMFS-η for the selection of crucial features subset to reduce high dimension in the original data set. They found that the classifier diagnoses with 95% accuracy [41]. Hakim et al. proposed an SVM (support vector machine) classifier for predicting COPD patients. Prediction of the model for the risk of 30-day readmission in a hospital with an experimental accuracy of more than 89% [42]. Amalakuhan et al. proposed a method using a random forest (RF) classifier to predict which patients were at huge risk for various COPD exacerbations and re-admission in a hospital in a single year. They performed different measurements to check the robustness of the model for this purpose. They calculated AUC, (NPV, PPV) negative/positive, sensitivity, and specificity. The AUC score was 0.72, PPV was 0.7 and NPV was 0.63, specificity was 0.56, sensitivity was 0.75 [43]. Badnjevic et al. proposed a method using fuzzy rules and an artificial neural network (ANN) to classify COPD patient’s lung function. So, for this attempt 285 COPD patient’s data were used, and they found 92% accurate classification [44]. Barúa et al. proposed a method using a feedforward artificial neural network (ANN) to classifying the patients who were affected by central and peripheral airways. For classification, they used 131 patients’ data set and the author found 98.47% correct classification. However, when the performance was examined with unseen data the classifier showed very poor results and it only acquired 61.53% accuracy [45]. Orhan Er and Feyzullah Temurtas proposed a method to diagnose COPD patients with the help of a multilayer neural network (MLNN) with two different structures of the neural network. The first structure consisted of only one hidden layer, and the second structure consisted of two hidden layers in MLNN. They used the backpropagation (BP) method with momentum. Levenberg marquardt (LM) classifiers were used for the training of the MLNN. The results showed 93.14% with one hidden layer for the BP algorithm and 94.46% result using FFNN along with LM with two hidden layers [46]. Fernandez-Granero et al. proposed a method for early detection of acute exacerbation of COPD (AECOPD) using principal component analysis (PCA) and along with support vector machine (SVM) and tried to improve the feasibility of computerized analyses for early detection of AECOPD patients. The system was able to predict with 75.8% accurately and exacerbations were disclosed with an average of 5 ± 1.9 days in early at medical attention [47]. Işık et al. proposed a method using an artificial neural network (ANN) to detect the four different stages of the COPD patient’s disease levels, i.e., the first one is mild stage, the second one is moderate, the third one is severe, and the fourth one is a very severe condition. The ANN was developed with two hidden layers and five layers for the cross-validation technique. Data collected from 507 patients and the ANN model showed high performance with the patient’s dataset. They conducted performance measures, such as MSE values 0.00996 and MAE values 0.02478, respectively [48].

Swaminathan et al. proposed a method for early discovery of the exacerbation of COPD patients. For this purpose, the author applied different classification techniques namely, support vector machine with polynomial (SVMP), with linear (SVML), with Gaussian (SVMG), RF, Naïve Bayes (NB), logistic regression (LR), KNN, and gradient boosted decision tree (GBDT). The results of all classifiers were compared, but the author found that only LR and GBDT showed better performance. The LR classifier showed 89.1% accuracy and the GBDT classifier showed 88.1% accuracy [49]. Yang et al. built three machine-learning models and compared the prediction of all the models, i.e., gradient boosting machine (GBM), regularized logistic regression (LASSO), and multi-layer perception (MLP). After that, the author used these methods to predict the risk of re-admission of COPD patients in the next 30 days. They used AUC for the measurement of model performance. They found GBM model accuracy 0.706, LASSO model accuracy 0.700, and MLP model accuracy 0.705 [50]. Raghavan et al. proposed a method using a combination of eight factors of the CAT (COPD Assessment Test) with other well-known factors of COPD (smoking history, age, and post-bronchodilator spirometry). For this purpose, they developed two models. The first model was stepwise logistic regression. This model was used to identify the relevant variable and the final model logistic regression showed moderate accuracy. They calculated measurements in form of AUC and found an AUC score of 77% [51]. None of the aforesaid research studies included information about the exacerbation of COPD patients. A summary of the literature review is shown in Table 1.

Xia et al. proposed a method using SVM with recursive feature elimination for the selection of relevant features. They selected nine features for the classification of COPD patients, to address the imbalanced classes. The SMOTE technique was used for oversampling of the data. For this purpose, 15 and 191 subjects in managed and control group data were used for classification. They found an AUC score of 0.987, an F1 score of 0.978, and a positive predicted value of 66.7% [52]. In Reference [53], they used 22 attributes for statistical analysis. They selected 20 independent prominent attributes (e.g., smoking, age, forced expiratory volume one, pulse, cough, and breath shortness) and two dependent features for the clinical decision to diagnose COPD and asthma patients. For this attempt, 132 samples were used with 22 attributes, they applied different classification techniques, but random forest classifier (RF) showed a precision value of 97.7% for diagnosing COPD patients. In the classification of asthma, RF showed 80.3% precision.

The previous studies show that many researchers suggest a different approach to diagnose COPD patients in different ways. Many researchers investigated the feature set and tried to highlight the importance of the feature set, following their research work. This is an individual study to classify COPD patients with a specific feature dataset.

## 3. Methods and Materials

### 3.1. Study Design and Subjects

This study is a cross-sectional, multicenter observational study. It was carried out at Haeundae Paik Hospital, Korea. This study was approved by the institutional review board with IRB No. 2020-03-007 for Haeundae Paik Hospital, and all the participants gave their consent to participate in this study.

### 3.2. Data Collection and Experimental Procedure

In this study, we used real-life data, and data were collected from 8 March 2012 to 31 December 2019, at Inje University Paik Hospital, Busan, Korea. The dataset contains 2900 patients suffering from COPD who were enrolled during this date. The dataset contains two classes: one is mild patients, and the other is severe patients. Moreover, the data were processed and analyzed, using a system with the following specifications: Windows 10, 3.60 GHz 64-Bit Intel Core i7-7700 processor, 24 GB RAM, Python 3.6.9, and TensorFlow 1.14.0, manufactured by intel and sourced from Gimhae, Korea. The complete algorithm for the proposed framework was developed in our lab, using the above specification.

### 3.3. Feature Engineering

In feature engineering, it is very crucial to select an important subset of features and removing unnecessary features that have the least effect on the performance, and obtain the excellent performance of a given ML classifier task. A small number of efficient feature subsets is more important for the construction of the classification model, and these subsets decreases the chance of the model having an overfitting problem.

Furthermore, big datasets require great computing power capacity and vast volumes of storage, and commonly generate the lowest classification accuracy. Feature selection is more important to select a good subset of features in many fields including finance, production, manufacturing, medicine, image processing, and biology. The recursive feature elimination (REF) is a technique for the selection of the best subset of optimal features, in the past study many researchers investigate and used [54,55,56,57,58,59,60]. In this study, we used the recursive feature elimination (RFE) technique to select the optimal feature subset for the classification of COPD patients. 54 features were collected for this study. Out of 54 features, 30 features were selected using the RFE technique and out of 30 features, it was reduced to 24 after consulting with the expert physicians in this field. The descriptions of the selected features are shown below in Table 2. 

### 3.4. Machine-Learning Algorithm and Evaluation Metrics

Machine-learning models are very effective for the classification of patients. The mild and severe patients of COPD can also be identified by using machine-learning classifiers, and the severity of the patients can be detected accurately. We developed five state-of-the-art ML classifiers, namely random forest (RF), support vector machine (SVM), gradient boosting machine (GBM), XGBoost (XGB), and K-nearest neighbor (KNN). However, the classification of COPD patients’ accuracy of individual classifiers was not ideal; no individual classifier got the ideal result, because the different classifiers have their parameter value and learning ability.

**Figure 1:**
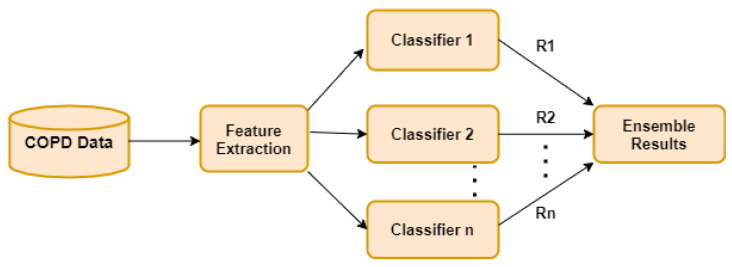
The basic architecture of SVE classifier for COPD classification.

We need to fine-tune some learning parameters according to the classifiers we use. Subsequently, the classifiers examine the extracted features to construct a classification model. There are certain limitations during the implementation of the classifiers. So, to avoid the limitation, the soft-voting ensemble approach (which is a combination of classifiers) was introduced by many researchers. The basic architecture of the soft voting ensemble (SVE) for the classification of COPD is shown above in Figure 1. In the development of machine-learning classifiers, hyperparameters are used to make an efficient and robust model. All hyperparameters are shown in Table 3 below.

Hyperparameters tuning is a method that is used to improve the performance of the model and also to optimize the cost of function. So, initially, to select the accurate set of hyperparameters, several iterations are performed by choosing the particular hyperparameters, using a 5-fold cross validation method mentioned above, in Table 4. However, we built six state-of-the-art classifiers, including five base classifiers, and the sixth is an ensemble classifier and trained them with extracted features. The model performance can be evaluated based on performance metrics parameters, such as accuracy, precision, recall, f-measure, and AUC curve. In the current scope of the study, we can calculate the accuracy of the classifier, shown in Equation (1).

(1) $$accuracy=\frac{\mathrm{tp}+\mathrm{tn}}{\mathrm{tp}+\mathrm{tn}+\mathrm{fp}+\mathrm{fn}\;}$$

Precision and recall in this study are used to examine the performance of the model for each class. The precision expresses the ratio between the COPD patients who are truly identified versus all the COPD patients. Equation (2) shows the precision of COPD patients.

(2) $$Precision=\frac{\mathrm{tp}}{\mathrm{tp}+\mathrm{fp}}$$

The recall expresses the ability to find all COPD patients in the dataset, equation (3) shows the method to obtain the recall.

(3) $$recall=\frac{\mathrm{tp}}{\mathrm{tp}+\mathrm{fn}}$$

where tp = true positive, fp = false positive, tn = true negative, and fn = false negative.

The f-measure can be defined as the weighted average (i.e., harmonic mean) of precision and recall. Equation (4) shows the method to gain the f-measure. The f-measure can be explained as an interpretation between the predicted result and the actual result of COPD-affected patients.

(4) $$f-measure=\frac{2\times \mathrm{precision}\times \mathrm{recall}}{\mathrm{precision}+\mathrm{recall}}$$

Furthermore, AUC is used for all classifiers, with good AUC scores that show the better performance of the classifier for predicting individual label class. A high score makes sure the classifier is robust and better at distinguishing between all COPD patient classes. Furthermore, in the current scope of the study in the all-learning procedure 2900 COPD patient’s data were used. Data were split into an 80:20 ratio: 80% of data were used for training, and 20% for testing. This split was used for all the classifiers. These sets are identical for all the classifiers. One more thing may be needed to be addressed, therefore cross-validation with 5-folds also was performed to understand the generalizability of each classifier. According to the learning abilities and limitations, every classifier shows a good result, for instance, RF 87.2180%, GBM 90.2255%, XGB 88.0773%, KNN 86.3587%, and SVM 88.1847% accuracy for the classification of COPD patient’s classes. However, the soft voting ensemble (SVE) method performed well and provides 91.0849% accuracy, the best result as compared to others.

### 3.5. The Complete Framework of the Proposed Study

**Figure 2:**
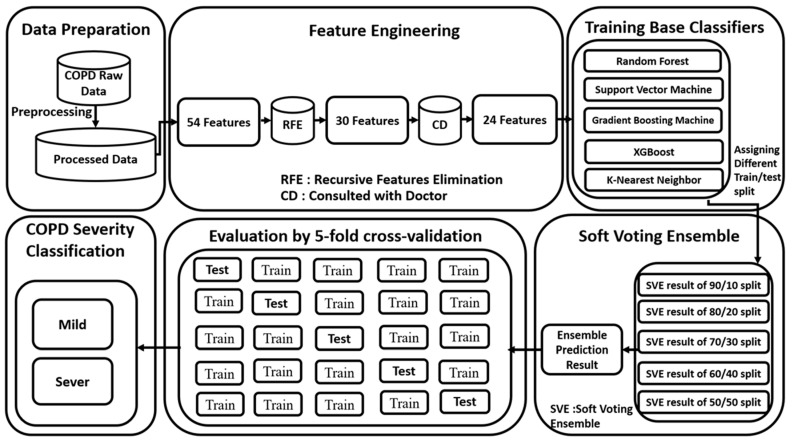
A complete framework of study for COPD patients for identification of different stages.

The complete procedure of the development of the system for COPD patients to detect the exact stage of the disease is shown in Figure 2. The entire flow of the experiment is divided into six basic parts, namely the data preparation, feature engineering, training base classifiers, soft voting ensemble, evaluation by 5-fold cross-validation, and COPD severity classification. In the feature-engineering part, two different steps were performed: in the first step, the most relevant features were selected, and in the second step, we consulted with the doctor. After consulting with a doctor, we removed some redundant features that are not valuable. In the third step, six state-of-the-art machine-learning models were developed, namely random forests classifier, support vector machine, gradient boosting machine, XGBoost, and K-nearest neighbor classifier. All the ensemble techniques consist of three things: diversity, training of base classifiers, and a combination of the prediction results of base classifiers. Diversity of an ensemble method defines that all machine-learning base classifiers must be individual as much as possible and their learning ability and decision boundaries should be dissimilar from each other. The use of different machine-learning classifiers with numerous parameter boundaries is an approachable method to better the diversity of an ensemble method. All of these machine-learning-based classifiers are trained with selected features so that they can generate different prediction models with their learning ability and decision boundaries with the same input data. It makes sure that every classifier generated numerous prediction models beneath their decision boundaries and learning ability with training parameters.

There are many competing machine-learning classifiers for training, but we used five of them to develop an ensemble model. The hyperparameters tuning technique was used to increase the performance of each classifier, for selecting the right set of parameters, the 5-fold cross validation method was used. Combining base classifiers combines the prediction result of individual classifiers, using an ensemble method.

There are numerous approaches for combining the results, but we selected the most widely used weighted average of the soft voting ensemble technique.

## 4. Results

**Figure 3:**
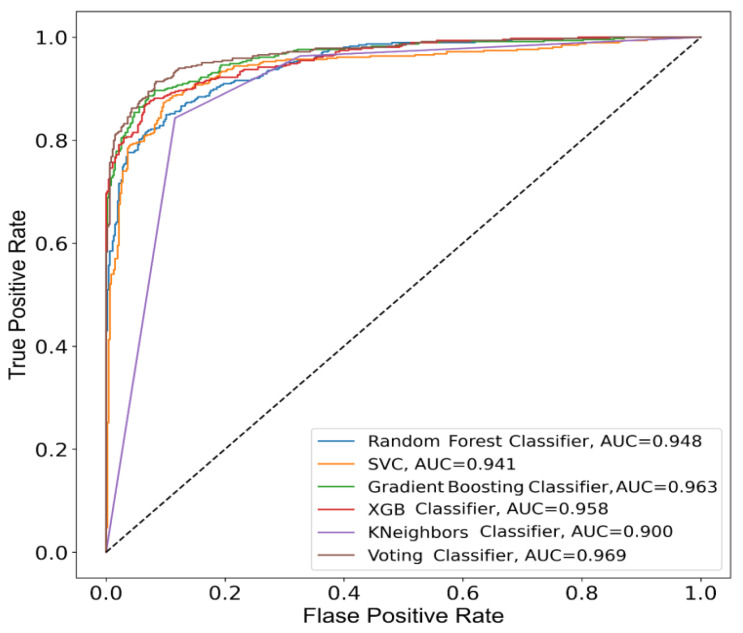
ROC–AUC curve of the proposed SVE method and the five classifiers.

The machine-learning classifiers implemented in this research study gave some valuable results in the terms of determining the right stage of patients suffering from COPD. The comparative analysis of different machine-learning classifier’s precision and with other measurements is shown in Table 5. The features and hyperparameters which are used in five proposed machine-learning classifiers for the ensemble method were shown in Table 2 and Table 3, respectively. It can be observed that the SVE method performed well in terms of classifying COPD patients. The ROC–AUC curve of the proposed SVE method and the five classifiers that are used in the ensemble method are shown in Figure 3. It was observed that the SVE method demonstrated the best generalizability in terms of forecast the data according to several test sets. Table 6, below, shows the overall machine-learning classifiers evaluation consequence of all classifiers. The reported performance of the classifiers mentioned in Table 5 and Table 6 was obtained by using test sets.

Moreover, the confusion matrix consists of two labels: label 0 represents the mild stage in which 466 patients were tested, with 423 correctly predicted and 43 incorrectly predicted; and label 1 represents the severe stage, in which 465 patients were tested, with 425 correctly predicted and 40 incorrectly predicted.

## 5. Discussion

**Figure 4:**
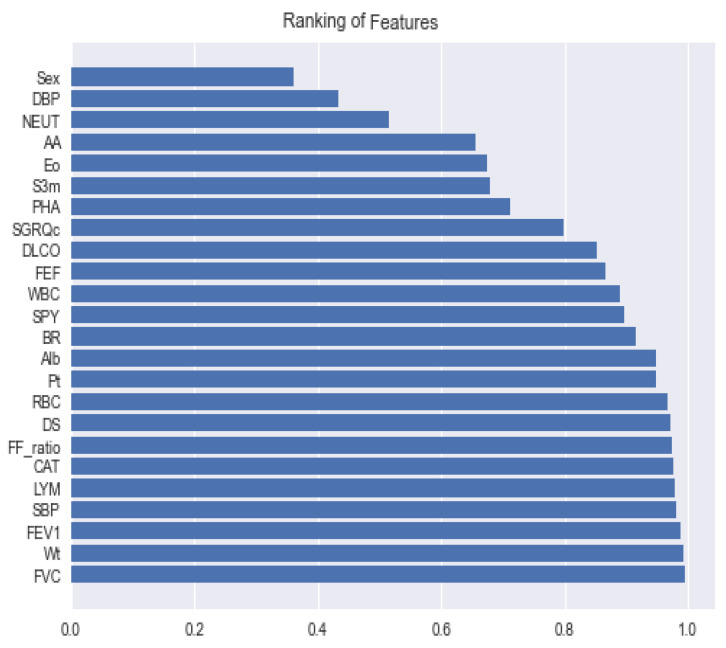
The features importance of the proposed soft voting ensemble classifier. Note that FVC, Wt, FEV1, SBP, LYM, CAT, FF_ratio, DS, RBC, Pt, Alb, BR, SPY, WBC, FEF, DLCO, SGRQc, PHA, S3m, EO, AA, NEUT, and DBP denote forced vital capacity, weight, forced expired volume in one-second prediction, systolic blood pressure, lymphocytes, COPD assessment test score, FEV1/FVC ratio, duration smoke, red blood cells, platelets, albumin, breath result, smoke per year, white blood cell, forced mid-expiratory flow, diffusing capacity of the lung for carbon monoxide, the St. George’s Respiratory Questionnaire, history of asthma, sputum3m, eosinophils, availability of asthma, neutrophil, and diastolic blood pressure, respectively.

The aim of this study was to develop a system that diagnoses the stage of severity of disease in COPD patients in a precise manner. For this purpose, the most crucial 24 features were selected and used for training. The importance (ranking) of the features is given below, in Figure 4. After that, we applied five different machine-learning classifiers, and at the end, the prediction results of all classifiers were integrated, using the soft voting ensemble method. Our proposed method for the classification of COPD got a significant result with an accuracy of 91.0848% and an AUC score of 96.8656%.

This study developed a framework to enhance the monitoring and treatment based on a decision tree for detecting the mild and severe stages for monitoring COPD patients. Due to imbalanced classes, the under-sampling method is used to manage the classes. The data were divided into two groups, mild and severe, and the most important features were selected, including medical history, vital signs, and various inflammatory and comorbidities indicators, that were then fed as input to machine-learning classifiers [35]. The relevant feature prediction result was better than the previous result for detecting the exacerbation in COPD patients. In previous studies, they have done similar work but using other feature selection methods to select relevant features. AUC curve was used to measure the performance of the model, and their AUC score was low as compared to our study [61]. In the previous study, many researchers have revealed the importance of features used for the detection of COPD exacerbation. The spirometry test values are also used to find the disease stage of COPD patients. In this work, they have used the feature named “FEF” for detecting COPD that could be an earlier marker rather than other markers, such as FEV1, DLCO, and FVC. One of the aims of this work is to determine the value of FEF at baseline and using that value the development of COPD disease could be predicted for the future 10 years or not and observed that even after some adjustment of smoking history, age, and FEV1/FVC the FEF is an independent risk factor for COPD at baseline [62].

The analysis shows that, in stable COPD patients, albumin concentrations were lower as compared to non-COPD. The albumin supports the existence of a deficit in systemic malnutrition, antioxidant and anti-inflammatory defense mechanisms in COPD [63]. The increase in the odds ratio is related to the WBC (white blood cell) quartile: if the WBC quartile is high, then the odds ratio is high. However, the odds ratio and WBC quartile do not have a significant influence on COPD and asthma [64]. Primarily, the guidelines for COPD focus on the prevention of weight loss. On the other side, the milder-stage patients with COPD are associated with obesity and overweight [65] and also in the global initiative for chronic obstructive lung (GOLD) that gives evidence for the diagnosis, treatment, and assessment of COPD that focus on the prevalent of weight loss [66]. The platelet increases in stable COPD patients as compared to control subjects. During the acute exacerbation of COPD, the platelet activation more increases [67], and this work also found that during the exacerbation platelet are increase [68]. The systolic blood pressure (SBP) and diastolic blood pressure (DBP) and pulse rate significantly are different in COPD patients as compared to in the manage group. The only pathophysiology can understand its complications [69]. The red blood cell distribution width (RDW) values were significantly higher in COPD patients as compared to the control group. Chronic obstructive pulmonary disease associated with patients with a risk of cardiovascular disease could be developed [70]. The most prevalent risk factor of respiratory disease in adults is cigarette smoking, and this disease is associated with airflow obstacles, so it is called emphysema and chronic bronchitis, typically known as a chronic obstructive pulmonary disease (COPD). Smoking is associated with the progress of chronic airflow obstacles, without asthma present. The airflow obstacles in current smokers create a 4.5 times higher risk as compared to those who never smoke [71]. There have been studies that have shown the importance and the purpose of features. Now, in the current study, the additional features could be helpful to explore the condition of patients who suffer from COPD. The literature survey revealed that the aforementioned different features have been widely used in the COPD analysis, and therefore also considered in the proposed method. Chronic obstructive pulmonary disease symptoms vary between individuals and show conflicting clinical presentations.

In recent years, machine-learning approaches have been emerging, and they give the possibility of defeating this limitation. Moreover, we checked the performance of our proposed model with different split ratios in the training and testing dataset. The performance of the model with the different split ratios is shown in Table 7.

Our proposed study was compared with numerous state-of-the-art models that used clinical features for differentiating the “mild” and “severe” stage of patients who suffered from COPD. The developed system in this proposed study was found to outperform all the former studies that use COPD-related classification. A comparative analysis of these proposed studies is shown in Table 8.

## 6. Conclusions and Future Work

In this study, we proposed an ensemble method to seek the severity of disease in patients who are suffering from COPD. For this purpose, twenty-nine hundred patients’ data were used, in which there were two classes: one class belonged to the mild-stage and the other belonged to the severe-stage patients. In the dataset, we had a total of fifty-four features. We then selected the most relevant features, using the RFE technique; after selecting features, we consulted with a doctor and reduced more redundant features, and only twenty-four were left. To alleviate the problem of imbalance in the training dataset, we used a robust and effective SMOTE method. Subsequently, we applied five state-of-the-art machine-learning classifiers, namely random forests, support vector machine, gradient boosting machine, XGBoost, and k-nearest neighbor. The soft voting ensemble, or weighted averaging approach, was used, and the prediction results of each classifier were combined for COPD patients, generating a final ensemble result of classification. Our proposed ensemble model result outperformed as compared to other individual classifiers and the former proposed methods. This research work is unique in the case of a combination of statistical features that is fed as input to the machine-learning classifiers. The proposed ensemble model performance with two stages of COPD patients’ measurements with an accuracy of 91.0849%, precision of 90.7725%, recall of 91.3607%, f-measure of 91.0656%, and AUC score of 96.8656, respectively, for classifying the mild and severe groups of patients. The ensemble method also alleviates the issues of over-fitting and under-fitting during the training and validation. Therefore, it turned out that our proposed SVE (soft voting ensemble) method was better than that of using an individual machine-learning classifier in COPD patients to distinguish the different stages of the disease.

We will collect more data for future studies and also will be dealing with more than two classes, aiming to address the multiclass problems related to COPD patients. Furthermore, we will try deep-learning models to improve the performance metric. From the current performance of our model in this research work, it is recommended that the implemented ensemble model could perform well in hospital environments in real-time situations.

## Figures and Tables

**Table 1:** Related work.

Reference	Features	Classifiers	Outcomes	Performance Indices
[36]	Clinical	LSTM, ANN, SVM	92.86%	Accuracy
[38]	Clinical	DTF	75.8%	Accuracy
[39]	Clinical	ANN	More than 90%	Sensitivity, Specificity, AUC
[40]	Clinical	Naïve Bayes, SVM	87.8%	Accuracy
[43]	Clinical	RF	75%	Sensitivity, Specificity
[44]	Clinical	ANN	92%	Accuracy
[46]	Clinical	MLNN	94.46%	Accuracy
[49]	Clinical	GBDT, LR	89.1%	Accuracy
[51]	Clinical	LR	77.6%	Sensitivity, Specificity

**Table 2:** Features used in our study.

No.	Attributes	Value	Description
1	Sex	M/F	Male/Female
2	DBP	Numerical	Diastolic blood pressure (DBP)
3	NEUT	Numerical	Neutrophil (NEUT)
4	AA	Yes, no	Availability of asthma (AA)
5	EO	Numerical	Eosinophils (EO)
6	Sputum3m	Yes, no	You have had phlegm almost every day for more than three months a year. Is it?
7	PHA	Yes, no	History of asthma (PHA)
8	SGRQc	Numerical	The St. George’s Respiratory Questionnaire (SGRQc). Over the past year, you’ve had several respiratory symptoms. Have you experienced it?
9	DLCO	Numerical	Diffusing capacity for carbon monoxide (DLCO)
10	FEF	Numerical	The forced mid-expiratory flow (FEF)
11	WBC	Numerical	White blood cell (WBC)
12	SPY	Numerical	Smoke per year (SPY)
13	BR	Numerical	Breath Result (BR)
14	Alb	Numerical	Albumin (Alb)
15	Pt	Numerical	Platelets (Pt)
16	RBC	Numerical	Red blood cells (RBC)
17	DS	Numerical	Duration of Smoke (DS)
18	FF_ratio	Numerical	The ratio FEV1/FVC
19	CAT	Numerical	COPD Assessment Test (CAT)
20	LYM	Numerical	Lymphocytes (LYM)
21	SBP	Numerical	Systolic blood pressure (SBP)
22	FEV1	Numerical	Forced expiratory volume in one second (FEV1)
23	Wt	Numerical	Weight (Wt)
24	FVC	Numerical	FVC (forced vital capacity): maximum volume of air that can be exhaled during a forced maneuver

**Table 3:** Severity detection: classifiers and specifications.

Classifier	Specification
Random Forest	n_estimators = 500, random_state = 0, criterion = ‘gini’, max_depth = 15, min_samples_split = 5, min_samples_leaf = 5
Support Vector Machine	kernel = ‘rbf’, degree = 4, gamma = 7.9, C = 20, decision_function_shape = ‘ovr’, probability = True, random_state = 0
Gradient Boosting Machine	learning_rate = 0.1, n_estimators = 500, max_depth = 15, min_samples_split = 5, min_samples_leaf = 5, subsample = 1, max_features = ‘sqrt’, random_state = 10
XGBoost	random_state = 0, silent = False, scale_pos_weight = 2, learning_rate = 0.1, colsample_bytree = 0.4, subsample = 0.9, objective = ‘binary:logistic’, n_estimators = 500, reg_alpha = 0.01, max_depth = 15, gamma = 7
K-nearest neighbor	n_neighbors = 2, weights = ‘uniform’, algorithm = ‘auto’, leaf_size = 40, p = 2, metric = ‘manhattan’

**Table 4:** Five-fold cross validation.

Classifier	5-Fold Cross Validation (%)					
	1st Fold	2nd Fold	3rd Fold	4th Fold	5th Fold	Average
Random Forest	84.0268	83.8926	86.3087	88.9784	87.2311	86.0875
Support Vector Machine	87.1140	87.5167	89.1275	91.5322	88.1720	88.6925
Gradient Boosting Machine	88.8590	88.4563	90.7382	91.5322	91.1290	90.1429
XGBoost	84.9664	84.0268	87.5167	90.0537	86.4247	86.5976
K-nearest neighbor	84.4295	85.3691	87.3825	86.2903	86.6935	86.0329
Soft voting ensemble (SVE)	90.2013	88.1879	92.2147	93.6827	91.1290	91.0831

**Table 5:** Comparative analysis of classifiers with different stages of COPD patients (%).

Classifier	Disease Severity	Precision	Recall	F-Measure
RF	Mild	85.3360	89.9141	87.5652
	Severe	89.3181	84.5161	86.8507
SVM	Mild	89.2070	86.9098	88.0434
	Severe	87.2117	89.4623	88.3227
GBM	Mild	87.7263	93.5622	90.5503
	Severe	93.0875	86.8817	89.8776
XGB	Mild	89.3569	86.4806	87.8951
	Severe	86.8750	89.6774	88.2539
KNN	Mild	84.9484	88.4120	86.6456
	Severe	87.8923	84.3010	86.0591
SVE	Mild	91.3606	90.7725	91.0656
	Severe	90.8119	91.3978	91.1039

**Table 6:** Overall performance of all classifiers (%).

Classifier	Accuracy	Precision	Recall	F-Measure	AUC
RF	87.2180	89.9141	85.3360	87.5652	94.7875
SVM	88.1847	86.9098	89.2070	88.0434	94.0616
GBM	90.2255	93.5622	87.7263	90.5503	96.3192
XGB	88.0773	86.4806	89.3569	87.8952	95.8452
KNN	86.3587	88.4120	84.9484	86.6456	90.0259
SVE	91.0849	90.7725	91.3607	91.0656	96.8656

**Table 7:** Performance of all classifiers with recall (sensitivity) on different samples of training and testing splits.

Classifier	Different Division of Training Set (%) and Testing Set (%)					
	90/10	80/20	70/30	60/40	50/50	Mean ± STD
Random Forest	87.9167	85.3360	84.5222	84.3592	84.0260	85.2320 ± 1.5762
Support Vector Machine	92.3076	89.2070	87.2675	86.1490	84.3485	87.8559 ± 3.0497
Gradient Boosting Machine	90.0000	87.7263	87.6177	87.4747	86.5853	87.7822 ± 1.1592
XGBoost	90.9090	89.3569	89.5434	89.3289	88.4892	89.1192 ± 0.4288
K-nearest neighbor	91.071	84.9484	84.0599	80.8593	79.3822	84.0641 ± 4.5296
Soft voting ensemble (SVE)	94.5945	91.3607	90.7172	89.5288	89.0691	91.0540 ± 2.1799

**Table 8:** A comparison analysis of our results with state-of-the-art models’ work for stage detection.

Author	Objective	Accuracy (%)	AUC Score (%)
Our Work	“Mild”/“Severe” detection	91.0848	96.8656
Peng et al. [35]	“Mild”/“Severe” detection	80.3	80.3
Ryynanen et al. [60]	“HRQoL” detection	77	69

## Data Availability

Private dataset (i.e., COPD dataset for two groups mild and severe) is not available online. The data used in this study are available on request from the corresponding author.

## Abbreviations

COPD	Chronic obstructive pulmonary disease
SVE	Soft voting ensemble
ML	Machine learning
AI	Artificial intelligence
AUC	Area under curve
ROC	Receiver operating characteristic
